# Environmental Health and Safety Implications of the Interplay Between Microplastics and the Residing Biofilm

**DOI:** 10.1021/envhealth.4c00148

**Published:** 2024-11-25

**Authors:** Xiaohan Wu, Fei He, Xueran Xu, Leilei Wu, Jinyu Rong, Sijie Lin

**Affiliations:** † College of Environmental Science and Engineering, Biomedical Multidisciplinary Innovation Research Institute, Shanghai East Hospital, Tongji University, Shanghai 200092, China; ‡ Key Laboratory of Yangtze River Water Environment, Ministry of Education, Shanghai Institute of Pollution Control and Ecological Security, Shanghai 200092, China

**Keywords:** microplastics, biofilm, dynamic interplay, environmental behaviors, alteration of ecological risks

## Abstract

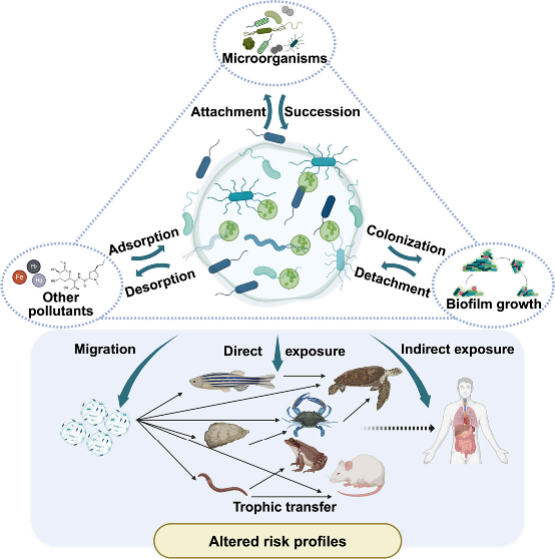

The increasing prevalence of microplastics in the environment has raised concerns about their potential environmental and health implications. Biofilms readily colonize microplastics upon their entry into the environment, altering their surface characteristics. While most studies have explored how biofilms influence the adsorption and transportation of other contaminants by microplastics, the reciprocal interplay between microplastics and biofilms and the resulting ecological risks remain understudied. This review comprehensively reviews the impact of microplastic properties on biofilm formation and composition, including the microbial community structure. We then explore the dynamic interactions between microplastics and biofilms, examining how biofilms alter the physicochemical properties, migration, and deposition of microplastics. Furthermore, we emphasize the potential of biofilm-colonized microplastics to influence the environmental fate of other pollutants. Lastly, we discuss how biofilm–microplastic interactions may modify the bioavailability, biotoxicity, and potential health implications of microplastics.

## 1 Introduction

Plastic, the century-old material, has taken center stage in the environmental health and safety sector in recent years due to its widespread presence in the environment and living species, including humans.^1^ The large annual production volume, the infiltration of plastic products in daily life, and the inadequate management and treatment of disposals have resulted in the ubiquitous distribution of plastics of various sizes, even reaching the equator and the Arctic.^2−4^ Although it might seem straightforward to collect and recycle bulk plastics, environmental weathering processes effectively break down plastics into smaller particles, i.e., micro- and nanoplastics,^5,6^ rendering monitoring and removal difficult.^7^ Additionally, plastics of different polymer compositions, shapes, colors, etc., add another layer of complexity to the problem.^8,9^ These intrinsic physicochemical characteristics of micro- and nanoplastics pose challenges in understanding their environmental fate and potential health risks.

Commendable efforts in delineating the toxicity potential and the corresponding mechanisms have shown that micro- and nanoplastics could cause oxidative stress, acute inflammatory responses, metabolic and neuronal disorders,^10−12^ and even death in organisms upon exposure. However, most current studies rely on virgin and spherical plastic particles, missing convincing and reliable environmental representations. Given the complex and dynamic nature of the environment, one would expect a constant change in physicochemical characteristics of micro- and nanoplastics as they interact with their surroundings. Specifically, the interactions between microorganisms and microplastics lead to a subsequent biofilm formation on the plastic surface, called the plastisphere.^13^ The microbial community that colonizes the microplastics differs from the surrounding environment, making them a new microbial niche compared to their counterparts in soil, rivers, lakes, and oceans.^14,15^ Moreover, the colonization of microbial communities could serve as potential carriers of environmental pollutants, altering conventional understandings of the environmental risks of the adsorbed pollutants and pathogens.^14,16,17^ Therefore, the colonization of microbial communities renders additional extrinsic characteristics to the microplastics, complicating the health implications of their hosts.^18,19^

The interplay between microplastics and the residing biofilms is dynamic. Initially, the composition and surface characteristics determine the colonization of the biofilms. The microbial strains on the surface of the microplastic vary during the biofilm’s maturity stage in response to community succession, a process also impacted by the microplastic’s characteristics and external factors.^20,21^ Over time, the biofilm modifies the properties of microplastics, including roughness, hydrophilicity, surface charge, specific surface area, etc.^22−24^ Understanding these dynamic changes and the rules that govern them is critical for assessing not only the environmental fate of microplastics but also their combined effects on the environmental health and safety profiles evolved from the interplay between microplastics and the residing biofilm.

Recent reviews shed some light on the occurrence of microplastics in different environmental media, the alterations in their properties due to biofilm formation, and their roles as carriers for other pollutants. It is important to note that the relationship between microplastics and biofilms is not unidirectional but a dynamic interplay between them. And the ecological risks posed by biofilm-colonized microplastics, rather than the toxicity of microplastics alone, remain insufficiently studied. We decided to focus on the bilateral interplay between microplastics and the residing biofilms. The effects of microplastics on the early formation and stability of biofilms as well as the structure of microbial communities are summarized, followed by a detailed description of the migratory transport and altered physicochemical properties of microplastics colonized by biofilms. The potential of biofilms to modify the vector characteristics of microplastics for chemicals and pathogens is also discussed. Overall, based on analyzing the different aspects of the interactions between microplastics and biofilm, we provide a systematic summary of the changes of microplastics with biofilms in bioavailability, bioreactivity, and human health effects. The aim is to offer new insights into the environmental fate and toxicological effects of microplastics as emerging contaminants and also perspectives for future research directions in this area.

## 2 The Interplay Between Microplastics and the Residing Biofilm

The interplay between man-made materials and biological systems has long been a research hotspot. Understanding the interactions enables researchers to develop biofriendly implants and create antifouling surface coatings for marine vessels.^25^ In the recent decade, the term “corona” has been used to describe the interactions between nanosized materials and proteins or biomolecules, a critical factor in determining the beneficial or detrimental effects of nanomaterials.^26,27^ Similarly, the term “plastisphere” was coined to describe the interactions between microplastics and environmental entities.^13^ While “corona” refers to the adsorption of molecules on nanosurfaces, the “plastisphere” encompasses a unique component: biofilms, a “living” biological community composed of heterotrophic, autotrophic, and commensal organisms.^28^ The dynamic and complex nature of biofilms makes the interplay between biofilms and microplastics particularly intricate. The following section summarizes biofilm formation and the factors influencing these interactions, highlighting how they alter the environmental behavior and toxicity of microplastics.

### 2.1 Effect of Microplastic Properties

**Figure 1:**
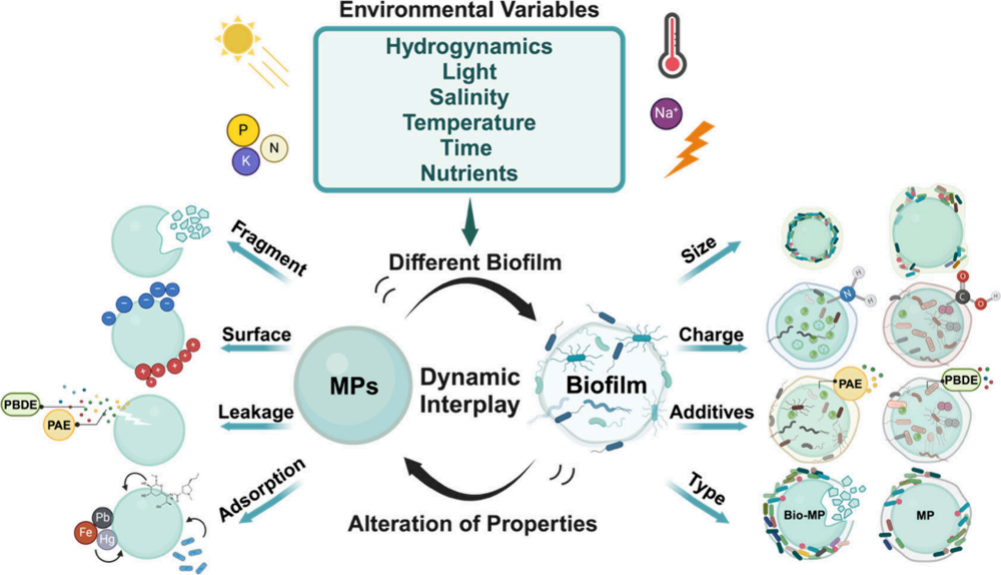
Dynamic nature of the interplay between microplastics (MPs) and biofilms.

The early stages of biofilm formation and development are significantly influenced by the physicochemical properties of microplastics,^29^ specifically during the conditioning layer phase (the initial biofilm) formed by extracellular polymeric substances (EPS). Factors influencing the early interactions between microplastics and biofilms include microplastic size, surface characteristics, biodegradability, and the presence of plastic additives (Figure 1).

Microplastic size significantly influences biofilm colonization due to changes in the specific surface area of the substrate to which microorganisms adhere.^30^ Compared to millimeter-scale microplastics, micrometer-scale microplastics are typically more easily adsorbed and colonized by microorganisms due to their larger surface area, providing more attachment sites and nutrient sources. In many cases, for microplastics larger than 100 μm, size primarily affects the formation rate and biomass of biofilms with a relatively minor impact on the distribution of microbial communities within the biofilm.^31^ However, when the size of microplastics becomes very small, less than 10 μm, the influence of size can manifest through changes in the composition of extracellular polymeric substances (EPS), thereby changing the structure of the biofilm.^32^ This imbalance arises from oxidative stress caused by smaller microplastics, which reduces the vitality of microorganisms and subsequently alters the protein:carbohydrate ratio within the biofilm EPS. Consequently, the structure of the biofilm becomes denser as the size of the microplastic decreases.

A recent study investigated variances in microbial community on biofilms of three distinct types of microplastics with the same size in marine ecosystems and found that polyethene (PE) demonstrated an elevated level of biomass compared to polystyrene (PS) and polylactic acid (PLA).^33^ This observation underscores that besides size, other characteristics of microplastics also vitally influence biofilm formation.^34,35^ Surface characteristics of microplastics, such as surface charge and hydrophilicity/hydrophobicity, also influence biofilm formation. For example, highly hydrophilic microplastic surfaces promoted the attachment and growth of aquatic organisms such as algae, while highly hydrophobic microplastic surfaces were more susceptible to bacterial colonization and hence biofilm formation.^36^ Hydrophobic microplastics like PE and PP tend to form biofilms more easily because their surfaces provide attachment sites and colonization environments suitable for microbial growth.^37^ Moreover, studies found that the production of *Spingomonas* in biofilms was closely related to the surface characteristics of the microplastics,^38^ preferring hydrophobic surfaces and degrading the microplastics to produce polysaccharides, thus facilitating biofilm formation.^39^ Further, the surface charge of microplastics plays an essential role in the early stages of biofilm formation (initial cell adsorption stage), and the hydrophobicity and roughness of microplastics are the most prominent parameters controlling the surface properties of microplastics, which can greatly influence the microbial community structure.^37^

Microplastics typically contain various plastic additives, such as plasticizers, antioxidants, and flame retardants. These additives might leach under photodegradation, weathering, or other aging conditions, forming a plastic leachate that affects the formation and colonization of biofilms on microplastics. Studies indicated that globally up to 23,600 t of organic carbon are leached from marine plastics annually.^40^ About 60% of it is available for microbial utilization in less than 5 days. Meanwhile, the leachate contains more labile compounds that are more bioavailable than natural organic matter, so it is more likely to promote biofilm growth.^41^ A study delineated differences in the stability of biofilm structure between low-density PE microplastics that incorporate UV stabilizers and those devoid of such additives.^42^ The results showed that the presence of UV stabilizers triggered the generation of intracellular reactive oxygen species within microorganisms, which augmented the proliferation of quorum-sensing mutants, ultimately culminating in the emergence of denser and more substantial biofilms. Moreover, heavy metals and organic compounds present in certain plastic additives can modify the composition and function of microbial communities, leading to a decrease in community diversity.^43^ These alterations may also impact the structure and function of the biofilms.

In addition to these points, the biodegradability of microplastics influences the selective attachment of microbes in the microplastic biofilms. For instance, a study compared the biofilms on the surfaces of biodegradable poly(3-hydroxybutyrate-*co*-3-hydroxyvalerate) (PHBV) and nonbiodegradable low-density PE, revealing that the biomass on the surface biofilm of PHBV was five times higher than that on PE.^44^ It indicates that biodegradable microplastics can degrade under microbial colonization, providing nutrients for microbial growth and forming a biofilm with higher biomass.^45^ In contrast, nonbiodegradable microplastics remain in the environment for extended periods, resulting in the formation of more stable and long-lasting biofilms. Consequently, the interplay between microplastics and biofilms not only modifies the properties of microplastics but also influences the microbial community and ecological processes. For example, a typical pathogenic bacterium, *Neisseria*, was enriched in PE biofilm and depleted in polyvinyl chloride particles (PVC), which might be due to the lower toxicity of PE than that of PVC.^46^

### 2.2 Effect of Environmental Variables

The dynamic interplay between biofilms and microplastics is intricately intertwined with environmental conditions, in addition to the influence exerted by the properties of microplastics.^24,45^ External environmental factors, such as hydrodynamic conditions, light exposure, salinity, temperature, time, nutrients, and external stresses, play crucial roles in biofilm development. Moreover, they have long-term effects on the overall colonization process and microbial community succession.

**Figure 2:**
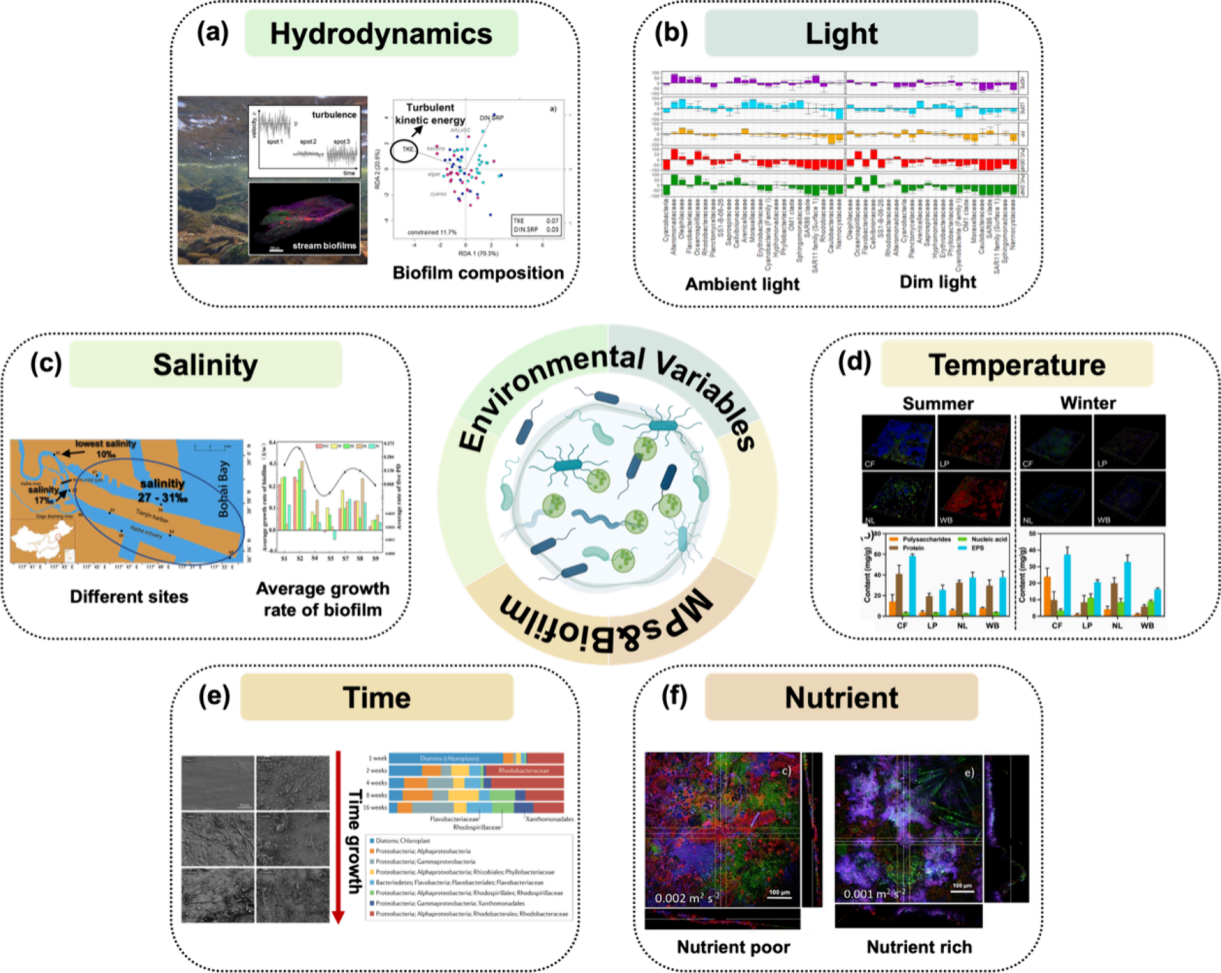
Effects of environmental variables on the interaction between microplastics and biofilms. (a) Turbulence affected the microbial composition of the biofilms. Reproduced with permission from ref (47). Copyright 2017 Elsevier. (b) The composition of species on microplastic biofilms was different under different light intensities. Reproduced with permission from ref (20). Copyright 2019 Pinto et al. (c) Variations in water salinity can affect the growth rate of biofilms. Reproduced with permission from ref (49). Copyright 2019 American Chemical Society. (d) Biomass and EPS content of microplastic biofilms is higher in summer than in winter. Reproduced with permission from ref (51). Copyright 2024 Elsevier. (e) As the biofilm on microplastics grew over time, the richness of the microbial community increased. Reproduced with permission from ref (55). Copyright 2020 Springer Nature. (f) Nutritional conditions can influence the specific glycoconjugates of the matrix EPS and the viability of bacteria on biofilm. Reproduced with permission from ref (47). Copyright 2017 Elsevier.

Natural environmental factors, including hydrodynamic conditions, light exposure, salinity, temperature, time, and nutrients, significantly impact the colonization of biofilms on the microplastic surface. Hydrodynamic conditions can be considered as important factors shaping the composition and architecture of biofilms grown. Studies have shown that microbial biovolume and surface area covered by the biofilm increased with turbulent kinetic energy, while biofilm thickness and porosity decreased (Figure 2a).^47^ Moreover, light plays an important role in the growth of biofilms on microplastics. Research indicated that the morphology and structure of biofilms varied across different seawater exposure depths, with these differences mainly attributed to variations in light intensity.^20^ As water depth increases and light availability decreases, both the total biofilm quantity and its thickness reduce, as illustrated in Figure 2b. Salinity predominantly influences both the microbial community diversity and the growth rate of biofilms on the surface of microplastics. Studies indicated that the microbial community composition and species richness on freshwater microplastics–biofilms exhibit significant differences compared to those on marine microplastics–biofilms.^48^ Additionally, a comparative study on biofilm growth rates in different environments revealed that biofilms on freshwater microplastics develop at a higher average rate than those in seawater, suggesting a potential negative correlation between biofilm growth rate and salinity (Figure 2c).^49^

Higher temperatures within an ideal range may cause faster growth and multiplication of microorganisms adhered to the surface of microplastics, as temperature and enzyme activity rates are closely associated.^50^ Microbial community structure may be affected by seawater temperature; microbial community diversity is relatively enriched at high water temperatures, as shown in Figure 2d.^51^ Seasons with higher temperatures, such as summer and spring, have more chlorophyll in their microplastic biofilm than autumn and winter, likely due to the optimal growth of most photosynthetic bacteria at 20–30 °C.^52,53^ In addition to the time of year (seasons) that influences the microbial communities developed on plastics in aquatic environments, time also affects community composition during the first hours and days after the plastic is immersed in seawater. Studies have shown that the diversity and structural complexity of the community increased over time from 0 to 16 weeks and that the dominant species varied at different times.^54^ Additionally, although plastic properties affect biofilm plantings, communities may become similar or converge over time as the life cycle progresses (Figure 2e).^55^ Furthermore, increased nutrient concentrations can decrease bacterial diversity while exerting a regulatory influence on the community. Diverse nutrient conditions also shape variable biofilm structure and composition (Figure 2f).^47^ This regulatory effect fosters a higher prevalence of bacterial groups such as *Sphingobacteriaceae*, *Cyanobacteria*, and *Alphaproteobacteria*.^56^

External environmental pressures, such as heavy metal ions and organic pollutants, also influence the development of biofilms on microplastics. Certain heavy metal ions can alter the composition and diversity of the microbial community within the biofilms. For instance, some bacteria in biofilms are susceptible to zinc ion toxicity.^57^ The structures of microbial communities in microplastic biofilms vary with different heavy metal ions. In a comparative study examining the effects of Pb(II) and Cu(II) on microplastic biofilms, Cu(II) at a concentration of 19.8 μmol/g in the biofilm enhanced microbial diversity, whereas 26.7 μmol/g Pb(II) had the opposite effect.^58^ This difference may arise because Cu(II) can serve as a nutritional element for some microbes, while Pb(II) can inhibit the growth of certain microorganisms due to its toxic effects.^59^ Research has also investigated the colonization of microplastics by biofilms under stress induced by sulfamethoxazole and tetracycline antibiotics. The presence of organic pollutants was found to influence the abundance of antibiotic-resistant bacteria on microplastic biofilms, thereby influencing the formation of the plastisphere.^60^

In summary, the size, surface properties, additives, and degradation properties of microplastics primarily influence the initial stage of biofilm formation. In addition to the influence exerted by the properties of microplastics, this dynamic interplay between biofilms and microplastics is intricately intertwined with environmental conditions. In general, factors that bolster the overall expansion of microbial communities, such as ample nutrition and appropriate temperatures, contribute to an increased growth rate of biofilms.^56^ However, rapid colonization and maturation of dominant species can lead to a reduction in the overall bacterial community’s diversity. Conversely, selective growth factors such as salinity and additional pollutants tend to increase microbial community diversity.^56,61,62^ These selective factors exert inhibitory effects, affecting the growth rate and biomass of biofilms, but their intervention introduces a larger array of bacterial populations within the biofilm, consequently enhancing the overall diversity.

## 3 The Dynamic Nature of the Interplay between Microplastics and Biofilms

Biofilm formation on microplastics is a dynamic process characterized by reciprocal interaction between microplastics and biofilms. During the later phases of biofilm growth, changes in microbial strains uniquely demonstrate the dynamic nature of this interaction.^15^ The biofilm on the microplastic surface acquires dynamic features through the succession of microorganisms over time. Following biofilm formation, microplastic mechanical stability can be compromised by microbial enzymes, leading to fragmentation into smaller microplastics and potentially turning biofilms into sources of microplastics. Conversely, the aggregation of microplastics induced by biofilms may make biofilms play the role of sinks.^55,63,64^ Building on the previous section, which discussed the factors influencing biofilm formation on microplastics, this section aims to summarize how biofilms modify the characteristics of microplastics. Specifically, the adhesion of biofilms alters the physicochemical properties of microplastics (including both their substrate and surface properties), thus influencing the migration and deposition.^52^ Moreover, EPS on the biofilm affect the accumulation capacity of microplastics for other pollutants.^28,65^

### 3.1 Alterations of Physicochemical Properties of Microplastics by Biofilm Colonization

First, the biodegradation and biofragmentation induced by biofilm formation can alter the physicochemical properties of microplastics, including their size, density, roughness, hydrophobicity, and functional groups.^28^ Biofilm degradation of microplastics is evident through the coexistence of the grooves and cracks on the surface of microplastics and embedded microbes.^66^ As biofilms initiate degradation, microorganisms can alter the surface properties of microplastics using biologically derived enzymes.^67^ This results in a loss of mechanical stability for microplastics, leading to further fragmentation into smaller microplastics and even nanoplastics. For example, PS microplastics (250 μm to 2 mm) with biofilms on their surfaces after 120 days resulted in nanoplastics and microparticles ranging from 56 nm to 4.5 μm.^68^ Regarding density, laboratory studies have confirmed that the density of microplastics increases with the growth, impacting their migration behavior in the environment.^69,70^ It has been reported that the sinking speed of polyethene terephthalate (PET) and PS with biofilms increased by 1% and 4%, respectively.^69^ However, the effect of biofilm on microplastic density also varies depending on the biofilm’s composition and the type of polymer in different environmental conditions. In freshwater systems, PET and PVC, which have higher densities than water, showed increased density after biofilm formation, whereas biofilm attachment resulted in a decrease in the density of PP.^71^ Overall, biofilm attachment can alter the density and effective size of MPs, potentially affecting their migration behaviors and distribution characteristics.^52,66^

Additionally, biofilms can dramatically modify the surface properties of microplastics, such as roughness and hydrophobicity.^56,72^ One study found that the surface of MPs with biofilms showed various grooves, pores, and cracks, suggesting that biofilm growth may roughen the surface of MPs.^56^ Furthermore, the hydrophobicity and functional groups of microplastics change with biofilm growth. Fourier transform infrared spectroscopy (FTIR) spectra are frequently used to study the changes of functional groups after biofilm formation.^73^ By FTIR, the C=O groups were observed on the surface of PE microplastics colonized by biofilms,^20^ and C=O groups may indicate microplastic degradation.^74^ The increased abundance of hydrophilic C–O and C=O groups on the PE surface has been proposed to explain how biofilm formation reduces the hydrophobicity of microplastics.^20^ In contrast, researchers observed an increase in surface hydrophobicity on PET upon the formation of a mature biofilm, through the hydrolysis of the ester bond.^75^ Thus, changes in the hydrophilicity of microplastics due to biofilms are also influenced by the type of plastics.

In addition to changes in the physicochemical properties of the microplastic surface, biofilm formation may involve the leaching of additives from microplastics.^18,76^ Various additives (e.g., plasticizer, flame retardant, and stabilizer) are usually added to plastic products to improve or adjust their mechanical and chemical performance.^77^ Several studies have confirmed that heavy metals (e.g., lead and chromium), pigments, phthalate acid esters (PAE), polybrominated diphenyl ethers (PBDE), and other toxic additives have been detected in leachates from aged microplastics.^78,79^ These results suggested that the leaching of microplastics could occur through biological degradation, indicating that biofilms may accelerate additive leaching. Microorganisms in the biofilm can metabolize polymer additives, using additives as nutrients to promote the biofilm growth.^18,80,81^ In other cases, however, biofilms may slow the release of additives. Studies have shown that biofilm coverage slows down the mass transfer of hydrophobic organic chemicals, potentially acting as an additional mass transfer resistance.^80^ Recent studies have also found that biofilms may protect microplastics in the upper water layer from UV irradiation, thereby weakening the degradation of microplastics.^82^

### 3.2 Alterations of Migration Behaviors of Microplastics by Biofilms

The colonization of biofilms on microplastics significantly alters their volume and density, which, in turn, affects their movement and transport behaviors in the environment. Specifically, changes in buoyancy and settlement velocity were observed. Most microplastics without biofilm are positively buoyant and located at the seawater–air interface. However, as biofilms grow, the density and settling velocity of the microplastics increase, leading to a decrease in their buoyancy. For instance, the attachment of large organisms, such as hydras and cladocerans, can slightly influence the sinking speed of PS by accelerating it by 4%.^69^ This effect is particularly pronounced with high-density microorganisms such as diatoms.^83^ As the density increases, the buoyancy of biofilm-colonized microplastics decreases accordingly. Some researchers have observed that microplastics begin to sink below the surface and exhibit signs of neutral buoyancy after 3 weeks of biofilm colonization.^15^ Therefore, microplastics with biofilm gradually sink, with most eventually becoming part of seabed sediments.^28^

However, this does not mean that all microplastics will inevitably settle. The effect of biofilms on the buoyancy of microplastics is reversible and depends on the stability of the polymers in seawater.^84^ If microplastics are exposed to external forces or other factors, such as spillage after ingestion by organisms, the biofilm on their surfaces can be disrupted or even completely removed, restoring their buoyancy.^85^ Thus, the biofilm on the surface of microplastics controls the vertical migration in water environment to some extent.

The vertical migration of microplastics in seawater can be considered to be a dynamic cyclic process. As microplastics gradually form biofilms in seawater, they form agglomerates, reducing their buoyancy and increasing their settlement rate.^69^ Simultaneously, microplastics with biofilms are more likely to be ingested or filtered by fish and benthic organisms, as the biofilm triggers the olfactory search and foraging behavior of some marine organisms.^86^ When the biofilm is removed, microplastics return to a positively buoyant state and float upward until enough microorganisms regenerate on the surface. Nevertheless, due to the relatively slow removal rate of biofilms by marine organisms, most microplastics eventually sink to the seabed.

The previous discussion primarily covered the vertical movement of the microplastics. However, ocean currents and submarine circulation also play a role in the rapid horizontal spread of microplastics.^87^ Biofilm colonization can affect the migration rate of microplastics with ocean currents. As microorganisms attach to the microplastic surface, the density of the microplastic increases, and its migration rate decreases.^88^ Moreover, microplastics with biofilms tend to be deposited more rapidly.^89^ Therefore, to better understand the dynamic variation of biofilm-colonized microplastics, it is essential to consider spatial and temporal variabilities as well as between differences between various plastic polymers.

### 3.3 Alterations of Accumulation of Other Pollutants of Microplastics by Biofilms

Microplastics adorned with biofilms can act as carriers for additional pollutants, including heavy metals and organic compounds. After biofilm formation on the microplastic surfaces, the physicochemical properties of particles (i.e., size, surface roughness, surface charge, surface area, etc.) inevitably change.^24^ These alterations influence the adsorption capacity of materials, with factors like surface roughness, surface charge, and surface hydrophobicity playing significant roles.^90,91^ Consequently, biofilm formation further affects the adsorption behavior of microplastics, thereby modifying the fate of other pollutants in the environment.

Numerous studies have demonstrated that microplastics with biofilms in aquatic environments exhibit enhanced adsorption of pollutants from surrounding water, encompassing metal ions and organic pollutants.^58,92,93^ Additionally, microplastics with biofilms show higher affinities for pollutants compared to pristine microplastics,^92^ with the enhancement of adsorption capacity surpassing other influencing factors such as UV radiation.^94^ This enhancement can be attributed to the intricate composition of the biofilms on microplastics, which includes anionic functional groups (such as the carboxy group and phosphate group of cell walls), significantly promoting the adsorption of positively charged metal ion complexes.^90^

Furthermore, the adsorption mechanism of pollutants by microplastics with biofilms is multifaceted, involving physisorption, chemisorption, and biosorption.^24,92^ For example, researchers determined that Bio-PS exhibited the highest adsorption capacity, indicating that heavy metal adsorption to Bio-PS was multilayered and heterogeneous, as modeled by the Freundlich isotherm.^92^ Moreover, microplastics with biofilms may serve as carriers of trace metals. Studies have found that biofilms altered the adsorption kinetics of trace metals on microplastics, with the enhanced adsorption mainly attributed to its complexation with functional groups contained in biofilms, such as carboxyl groups and amino groups.^95^ These findings underscore the significant role of biofilms in microplastics in modifying the migration and fate of heavy metals and organic pollutants in the environment.

In addition to heavy metals, previous studies have revealed that microplastics with biofilms can also adsorb a wide range of organic pollutants, such as antibiotics, hydrophobic organic pollutants (HOC), polycyclic aromatic hydrocarbons (PAHs), etc.^66,96^ For instance, a study of microplastics in the Fei Lai Xia Reservoir in Guangdong Province, China, showed that the total adsorption capacity of 16 PAHs by microplastics ranged from 25.6–89.6 ng/g, indicating that the concentration of PAHs on biofilm microplastics is significantly higher than that found in natural waters.^97^ Moreover, it has been demonstrated that biofilms enhanced the adsorption of tetracycline by microplastics and promoted their stability.^98^ This will not only lead to the development of antibiotic-resistant bacteria but also inhibit the growth of sensitive bacteria.^99^ This selective pressure drives the evolution and acquisition of antibiotic resistance. Therefore, biofilms on the surface of microplastics are regarded as “hot spots” for antibiotic resistance genes, mainly manifested in the enrichment of resistance genes and their potential hosts, resistant bacteria.^46,63^ Thus far, researchers have found that microplastics with biofilms enrich multi-drug-resistant genes (sulfonamide-resistant genes) and mobile genetic elements (MGEs) that persist and propagate in the aquatic environment.^13,100^ A recent study revealed the effects of doxycycline (DOX) on the soil plastisphere, showing that DOX exposure increased the abundance of most *tet* genes on microplastics and the abundance of *intl1* associated with horizontal gene transfer (enhanced to 1.07 × 10^8^ to 8.37 × 10^7^ copies/g).^101^ Therefore, the presence of biofilms is critical when considering the actual environmental fate and risk of microplastics, and their interactions with other environmental contaminants cannot be ignored.

Although microplastics with biofilms demonstrate robust adsorption capacity for heavy metals and various organic compounds, this capability must also consider the variations induced by exposure to different environments and types of microplastics. First, the influence of different environmental media should be considered. A long-term exposure of PE microplastics in both water and soil environments showed microbial colonization and biofilm formation. Further analysis revealed that microplastics in soil exhibited a higher adsorption capacity for Cu(II) and tetracycline compared to those in water environments.^14^ Second, within the same environmental medium, such as an aquatic environment, biofilms colonized on microplastics demonstrated different adsorption characteristics in water bodies with varying salinity and nutrient elements.^34^

In addition, the type of contaminant also influences the altered role of the biofilm as a carrier for microplastics. Exposure of biofilm-free and biofilm-attached microplastics to a solution of nine organic emerging contaminants (ECs) revealed that most compounds exhibited 3.8 times lower concentrations on biofilm-attached microplastics than on biofilm-absent microplastics, with only a few compounds showing enhanced adsorption.^102^ The reason for these discrepancies may be that biofilms formed on the surface of microplastics under different external conditions have varying distributions and EPS compositions, resulting in different adsorption properties and affinity for heavy metals, antibiotics, and natural organic matter.^103^

The accumulation and dispersion of pollutants by biofilms on degradable plastics are also affected by the plastic degradation process, contrasting with more persistent plastics.^104^ The primary adsorption mechanisms include coordination/complexation of heavy metal and the EPS components on the colonized biofilm, surface complexation, and electrostatic interaction.^96^ Several studies have demonstrated that biofilm adhesion and degradation significantly enhanced the adsorption of heavy metals by polylactic acid (PLA) microplastics and polybutylene succinate (PBS) microplastics.^105,106^ It should be noted that EPS components produced by colonizing microorganisms can provide durable binding sites for the adsorption of pollutants due to their strong adhesion ability. In the presence of biofilms, contaminants adsorbed on microplastics are more difficult to desorb in the environment, posing a potential risk that cannot be ignored. Therefore, it is essential to consider the actual environmental characteristics when different types of microplastics are exposed and comprehensively assess the contribution of biofilm-colonized microplastics to contaminant migration.

In short, the formation of biofilms can lead to increased biodegradation by enhancing the secretion of intracellular and extracellular enzymes, causing changes in physicochemical properties, such as size and density variation, roughness alteration, and additive leakage. Within this, changes in the density and volume of microplastics can lead to a modified migration and deposition in the environment. Moreover, the ability of biofilm-colonized microplastics to accumulate toxic pollutants is influenced by extracellular polymeric substances (EPS) and attached microorganisms, which enhance the adsorption of contaminants, such as persistent organic pollutants, heavy metals, and antibiotics. This interaction creates a unique particle surface coating, representing the actual behavior of microplastics in the environment.

## 4 The Altered Risk Profiles of Microplastics Due to the Interplay with Biofilms

The interaction between biofilms and microplastics creates a distinct surface coating on the particles, representing their true environmental identity. While extensive research has been conducted on the exposure risks of model microplastics to humans and animals, less is known about the risks of microplastics in complex and dynamic environments. These biofilm–microplastics interactions can alter the ecological and health risks associated with microplastics. Biofilms change the exposure pathways of microplastics to organisms and modify the toxic effects of adhered microplastics. The following sections explore these changes in detail.

### 4.1 Changes in Organism Ingestion of Microplastics

**Figure 3:**
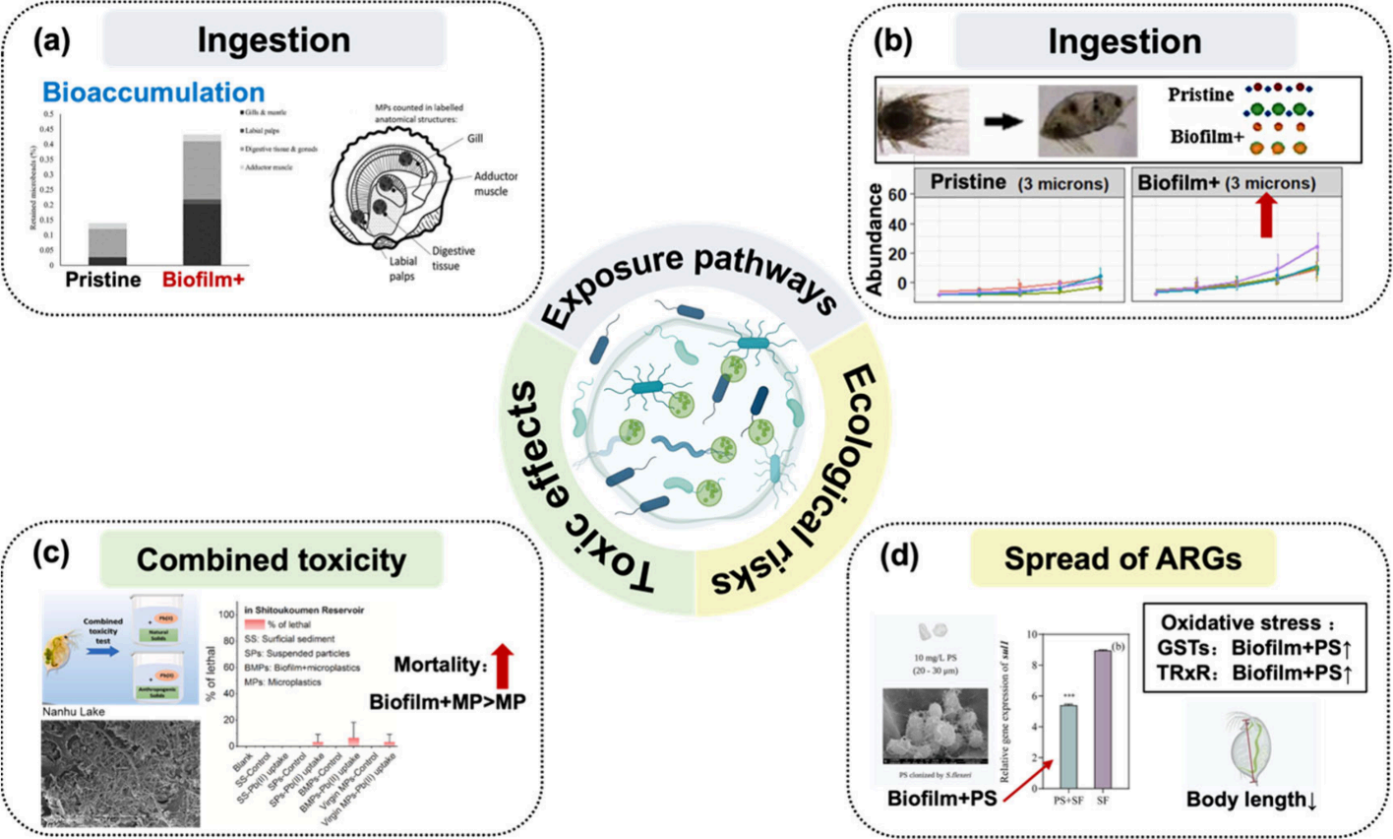
Impact of microplastic biofilms on toxicity and environmental health risks. (a) The concentrations of microbeads found in oysters exposed to biofilm-colonized MPs (42.360 ± 23.588 no/g) were higher than those exposed to virgin MPs (0.872 ± 0.317 no/g). Reproduced with permission from ref (19). Copyright 2021 Elsevier. (b) *Amphibalanus Amphitrite* ingested more biofilm+ PS (3 μm) than pristine PS (3 μm). Reproduced with permission from ref (109). Copyright 2022 Elsevier. (c) *D. magna* exposed to BMPs–Pb(II) exhibited higher mortality than *D. magna* exposed to Virgin MPs–Pb(II). Reproduced with permission from ref (59). Copyright 2021 Elsevier. (d) *D. magna* can acquire resistance genes and through microplastic biofilms and be affected by them in morphology and oxidative stress. Reproduced with permission from ref (155). Copyright 2023 Elsevier.

Biofilms significantly influence the ingestion and utilization of microplastics by organisms. Although many studies have investigated the ingestion of microplastics by biota and their transfer through food chains, the role of biofilms in these processes under real-world conditions is often overlooked.^19^ Studies have shown that organisms were more likely to ingest biofilm-covered microplastics than virgin microplastics, potentially increasing mortality rates.^107,108^ For example, oysters exposed to biofilm-covered microplastics ingested significantly more microbeads (42.3 ± 23.5 no/g) than those exposed to untreated microplastics (11.4 ± 0.6 no/g) (Figure 3a).^19^ This suggests that biofilm-covered microplastics may be mistaken for organic matter by filter-feeding organisms, thereby increasing the level of ingestion. This phenomenon was also observed in other aquatic feeders, such as fish, invertebrates, predators, and birds. Nousheen et al. found that biofilm-covered PS microspheres caused significant mortality and were more readily ingested by *Amphibalanus amphitrite* larvae (Figure 3b).^109^ Although microplastics did not bioaccumulate in the short term, ingested coated microplastics may be transferred to higher trophic levels. Once inside the human body, microplastics may pose health risks through accumulation or translocation, generating reactive oxygen species and activating antioxidant-related enzymes and mitogen-activated protein kinase signaling pathways.^107,110,111^

However, there is variability in the propensity and extent of ingestion of biofilm-colonized microplastics by organisms, which can be influenced by the size of the microplastics. For instance, *D. magna* exhibits a greater preference for ingesting pristine PS nanoplastics over biofilm-coated nanoplastics.^108^ Due to chemoreceptor effects, organisms ingested 3 μm biofilm-colonized microplastics at a higher level than 10 μm particles.^109^ Thus, biofilm colonization led to a nonuniform biotic uptake, reflecting different effects on various species. This variability is partly due to differences in microplastic properties, which are affected by the biofilm formation.

### 4.2 Changes in Toxicological Effects of Microplastics Induced by Biofilm Formation

Early studies on microplastic toxicity have shown various harmful effects on organisms and humans, including oxidative stress, cytotoxicity, acute inflammatory response, metabolism disruption, and neurotoxicity.^10,11,112^ The diverse physicochemical properties of microplastics and their interactions with other pollutants contribute to these toxic effects.^113,114^ Biofilm formation, which alters the physicochemical properties of microplastics, inevitably changes their ecological risks and toxic effects.

Biofilm formation induced degradation and the size reduction of microplastics. The colonization of biofilms was found to enhance the secretion of intracellular and extracellular enzymes,^56^ leading to the degradation of microplastics into smaller and even nanoplastics with higher toxicity.^18,115^ Studies on *Caenorhabditis elegans* showed that PS of smaller plastic particles were more likely to cause oxidative stress and perturb energy metabolism.^116^ This phenomenon parallels the size-toxicity effect exhibited by other particulate contaminants.^117^ Additionally, smaller microplastics were more likely to accumulate and migrate in the body after being inhaled by animals and humans, resulting in greater detrimental effects. A study on the distribution of PS during zebrafish development showed that small-sized PS accumulated in the yolk sac as early as 24 h postfertilization (hpf) and migrated to the gastrointestinal tract, gallbladder, liver, pancreas, heart, and brain throughout development (48–120 hpf).^118^ Smaller particles also have higher translocation rates and intestinal toxicity in complex models of human enterocytes.^119,120^ Disruption of gut homeostasis affects many physiological processes, including immune, nutritional, and metabolism functions;^121,122^ thus, the human health risks induced by the modification of biofilm on microplastic particle size cannot be ignored.

Biofilm formation can also influence the toxicity of microplastics by altering the functional groups on their surfaces. During the colonization of microplastics by biofilms, chemical reactions occur, introducing new functional groups such as −OH, −C=O, and −COOH, thereby altering their surface charges.^123,124^ The surface charge type is crucial as it governs the absorption, translocation, and toxicity of particles and notably impacts cellular responses in mammals. For instance, after oral administration of three different types of PS with different charges, positively charged microplastics more significantly affected the gut microbiota and intestinal barrier function in mice.^125^ Furthermore, oxidative stress is widely recognized as a toxic mechanism induced by microplastics with functionalized microplastics found to generate reactive oxygen species (ROS). Consequently, cells exhibit distinct antioxidant capacities when exposed to microplastics with different charge types. Thus, surface charge emerges as a critical determinant of cell death induced by the interaction between microplastics and cells, which is also the key mechanism through which biofilms alter the surface functional groups of microplastics, leading to charges in particle toxicity.^126,127^

Biofilm formation also plays a role in the release of potentially toxic substances during the destruction of microplastics. Microplastics contain a diverse range of chemical additives, such as bisphenol A (BPA), polycyclic aromatic hydrocarbons (PAHs), and polybrominated diphenyl ethers (PBDEs),^128,129^ which can induce immune responses, peroxisome proliferation, neurotoxicity, and alterations in gene expression profiles.^130^ For instance, organic carbon leached after the structural degradation of plastics was found to stimulate microbial activity in the ocean.^131^ Plant germination was adversely affected by leachates from microplastics, potentially linked to negative impacts on cellular energy metabolism systems.^132^ Biofilm-colonized microplastics disrupted the intestinal flora of *Enchytraeus crypticus* more than the original microplastics and reduced its reproduction rate. This effect was attributed to biofilm formation altering the leaching behavior of the microplastics.^133^ As a commonly used plastic additive, polychlorinated biphenyls (PCBs) were found to be closely related to liver cancer and developmental neurotoxicity through human inhalation and skin contact.^134^ In other cases, the presence of biofilms may slow down the release of plastic additives, reducing their potential ecological risks and achieving detoxification effects.^135^ Therefore, for a more accurate risk assessment of the migration and emission of plastic additives in the environment, it is necessary to thoroughly explore scenarios of plastic additive leakage induced by the colonization of microplastic surfaces by biofilms.

Biofilm-colonized microplastics can become carriers of heavy metals and other chemical pollutants, which have adverse effects on the environment and ecology and may also have negative effects on the human body through trophic transfer or direct exposure. These pollutants are associated with serious chronic diseases, including cancer, hormonal dysfunction, and other genetic problems in humans.^136^ In particular, the presence of biofilm can make it possible for heavy metals to hijack microplastics into organisms and humans. For instance, previous studies have demonstrated that biofilms enhanced the combined toxicity of both heavy metals and microplastics in *Daphnia magna* (Figure 3c)^59,137^ and also contribute to lower growth rates and survival rates of marine organisms.^138^ In certain cases, the physical damage caused by the ingestion of microplastics alone may be promptly repaired,^139^ whereas the presence and accumulation of heavy metals may make these adverse effects more persistent and irreversible. This was exemplified in a study where the combined exposure to microplastics and Cd^2+^ not only heightened Cd^2+^ bioaccumulation in *Euplotes vannus* but also worsened the reduction in ciliate biomass by increasing oxidative stress and causing membrane damage.^140^ Additionally, heavy metals adsorbed on microplastic biofilm can be absorbed into the digestive tract of the human body, where their prolonged presence can lead to health issues such as mood swings and cognitive impairments.^141^ Besides some common heavy metal ions, microplastic-associated biofilms have been reported to highly adsorb radioactive metal cations, which cause microplastic–biofilm consortiums to potentially emit radioactivity.^142^ Various studies have provided evidence that chronic exposure to radioactive emissions can lead to genetic mutations, cancers, hormonal disruptions, and brain damage, among other health issues.^137,143^

In addition to the toxicity brought by heavy metals and radioactive metals absorbed by biofilm-colonized microplastics, the coexisting organic pollutants could also modify their toxic effects. Data from 55 laboratory studies have demonstrated that microplastics in coexisting contaminant solutions, comprising both organic and inorganic contaminants, significantly exacerbate toxicity to fish.^144^ However, there is still debate about whether microplastics with biofilms that adsorb environmental contaminants result in higher or lower toxicity to fish than that caused by the pollutants alone. A study determined the diet assimilation efficiencies of PCBs absorbed to microplastics and food by goldfish and found that the level of microplastics was not directly proportional to the bioavailability of PCBs.^145^ This further suggests that the coexistence of microplastics and POPs may not increase bioaccumulation in all organisms. Therefore, further explorations are necessary to fully comprehend the inherent mechanisms of toxicity in microplastic–biofilm–contaminant complexes.

### 4.3 Changes in Environmental Health Risks from Biofilm-Colonized Microplastics Coexisting with Specific Pathogens

Biofilm-colonized microplastics may facilitate the spread of pathogens and antibiotic resistance genes (ARGs), thereby inducing associated environmental and health risks. On the one hand, biofilms adhering to microplastics serve not only as carriers for the accumulation and transport of pollutants but also as habitats for specific microbial communities (e.g., antibiotic-resistant bacteria (ARB)).^100,146^ The taxonomic structures of the bacterial communities colonizing the surface of microplastics in different environments significantly differ from those of the microbial communities in natural substrates.^34,147^ As mentioned in the previous chapter, microplastics can make the structure of microbial communities in water and soil environments more stable and may inevitably promote the colonization of their surfaces by some potentially harmful bacteria.^148^ Biofilms provide stable attachment sites for these microorganisms and protect them from mechanical damage and predation.^18,149^ In such scenarios, pathogenic microorganisms can utilize microplastics with attached biofilms as carriers to enter the tissues of organisms, evading immune system attacks and causing tissue damage.^16^ A study indicated that nearly half of the microbial populations on the biofilm of low-density polyethylene (LDPE) microplastics were potential pathogens such as the plant pathogen *Agrobacterium* and the fish pathogen *Flavobacterium*.^62^ Moreover, two opportunistic human pathogens (*Pseudomonas monteilii* and *Pseudomonas mendocina*) selectively colonize biofilms on MPs by comparison with biofilms on other natural surfaces (e.g., rock particles and tree leaves).^17^ In addition, once within the organism, pathogenic bacteria can disrupt the normal gut flora. Alterations in the gut microbiome can lead to adverse effects such as the proliferation of harmful species, increased gut permeability, endotoxemia,^150^ and reduced self-immune function.^151^

On the other hand, it was mentioned above that microplastic biofilms will enhance the adsorption of antibiotics, and the coexistence of antibiotics and microplastics leads to a higher risk of transmission of ARGs and MGEs.^152^ Through the analysis of microbial community composition and structure, it was observed that this phenomenon may be attributed to specific microorganisms selectively adhering to the surface of microplastics via biofilm formation. In essence, potential hosts of ARGs exhibit selective colonization of microplastics.^60^ Zettler et al.^13^ reported that the alpha diversity of bacterial communities on PE and PP biofilms was higher than in the surrounding seawater. Importantly, potential pathogenic bacteria such as *Legionella*, *Mycobacteria*, *Neisseria*, and *Toxoplasma* were enriched in microplastic biofilms, and these bacteria may serve as hosts for ARGs and MGEs.^153^

Moreover, the enrichment and dissemination of ARGs by biofilms on MPs significantly impact the ecological balance of microbial communities in the environment, consequently leading to the development and spread of antibiotic resistance.^154^ The related biological effects and toxicity risks arising from this are also of concern. Some researchers exposed *D. magna* to polystyrene microplastics colonized by biofilms and found that *D. magna* can acquire a sulphonamide-resistant antibiotic resistance gene through microplastic biofilms (Figure 3d).^155^ And these biofilm-colonized microplastics also disrupted the endocrine system of *D. magna* and altered the metabolism of ecdysteroids, thereby impacting their development and reproduction. Moreover, horizontal transfer of ARGs can occur among different bacterial taxa, rendering human pathogens susceptible to acquiring antibiotic resistance in biofilm-colonized microplastics. Consequently, the co-occurrence of microplastics and ARGs may expedite gene exchange among drug-resistant bacteria in the microenvironment of organisms and humans,^156^ thereby potentially fostering the transition from single antibiotic resistance to multidrug resistance. This can pose a potential threat to the health of organisms and humans. In particular, the spread of ARGs due to microplastics with biofilms may pose a serious health challenge to high-risk individuals in the elderly population, children, and immunocompromised patients. Future research on managing microplastic pollution in soil or aquatic environments can also focus on how to reduce the horizontal transfer efficiency of ARGs and the probability of potential pathogens acquiring antibiotic resistance by blocking the formation of microplastic biofilms.

In summary, the changes in health risks and toxic effects of microplastics due to their interaction with biofilms fall into two main areas. First, biofilm-colonized microplastic particles may be regarded as organic matter that is more readily ingested by organisms and accumulates and translocates within the body, which alters the risk of toxic exposure to microplastics. Second, the altered physicochemical properties of microplastics due to biofilm colonization may also result in toxic effects and mechanisms different from those of the virgin particles. Moreover, biofilm formation can enhance or attenuate the ecotoxicity of microplastics and other chemical pollutants (heavy metals, organic pollutants) and has the potential to spread pathogenic bacteria and ARGs, thus posing a risk to the environment and human health (Table 1).

**Table 1:** Altered Risk Profiles of Microplastics Due to the Interplay with Biofilms

type of plastics^a^	size	time	altered risk profiles	biological models	results	ref
PMMA	45 μm	14 d	changes in organism ingestion	*Ostrea edulis*	increasing uptake by a factor of ten	(19)
PS	91.26 nm	0–6 h	changes in organism ingestion	*Daphnia magna*	reducing EC_50_ and affecting feeding ability	(108)
PS	3 and 10 μm	0–5 d	changes in toxicological effects	*Amphibalanus amphitrite*	inhibiting the settlement and inducing precocious metamorphosis	(109)
PP, TWP	150 μm	7 d	changes in toxicological effects	*Enchytraeus crypticus*	disturbing the gut microbiota, decreasing the reproduction rate	(133)
PS	2 mm	0–6 h	changes in toxicological effects coexisting with Pb(II)	*Daphnia magna*	enhancing the combined toxicity	(59)
PS	1–5 μm	96 h	changes in toxicological effects coexisting with Cr(VI)	*Pomatoschistus microps*	decreasing the predatory performance (≤67%) and inhibited AChE activity (≤31%)	(138)
PS	0.22, 1.07, 2.14, and 5.00 μm	96 h	changes in toxicological effects coexisting with Cd^2+^	*Euplotes vannus*	increasing the bioaccumulation of Cd^2+^, decreasing the biomass of ciliate by increasing oxidative stress and membrane damage	(140)
PE	100–500 μm	5 d	changes in toxicological effects coexisting with PCBs	*Carassius auratus*	increasing the bioavailability of PCBs	(145)
PS	20–30 μm	7 d	changes in toxicological effects coexisting with ARB	*Daphnia magna*	altering the metabolism of ecdysteroids and transferring ARGs	(155)

a Note: PMMA, poly(methyl methacrylate); PS, polystyrene; PP, polypropylene; TWP, tire wear particles; PE, polyethylene.

## 5 Conclusion and Prospective

This review summarizes the environmental health and safety risks stemming from the interplay between microplastics and biofilms. It highlights the dynamic nature of these interactions, emphasizing how the inherent properties of microplastics along with various environmental factors shape their interplay. Biofilms not only modify the initial physicochemical attributes of microplastics but also influence their migration behavior and the accumulation of additional pollutants. These modifications ultimately affect the toxicity and the ultimate environmental fate of microplastics. Precisely, biofilm-colonized microplastics alter the biological exposure risks for organisms by affecting their ingestion. Additionally, the toxic effects and health risks posed by microplastics are further complexed by the accumulation of chemical contaminants and pathogens within the biofilms. The following recommendations outline further research directions in this field.

- Advancement in characterization methodology. Currently, most studies relied on characterization methods similar to those used for engineering materials to identify and analyze microplastics,^157^ including zeta potential measurement, electron microscopy, Fourier transform infrared (FTIR) spectroscopy, Raman spectroscopy,^158^ gas chromatography–mass spectrometry (GC-MS), etc.^159^ However, biofilm formation on microplastics upon entry into the natural environment or organisms has been relatively overlooked. The lack of biofilm characterization of microplastics may result in the loss of critical fingerprint data, such as their source and residence time in the environment or within organisms. Analyzing biofilms on microplastics could provide valuable insights into the fate and transport of microplastics in the environment.
- Understanding the dynamic interplay. Biofilm formation is influenced by the characteristics of microplastics and the surrounding environment. Most of the current studies focus on popular plastic types like PS and PP, while the everyday plastics received less attention. This review has shown that the modifications of the physicochemical properties and environmental fate of microplastics can vary dynamically depending on different factors, leading to increased uncertainties. Therefore, further investigation of microplastics released from plastic products across a wider range of spatial and temporal scales is required to gain a more comprehensive understanding of the interplay between microplastics and biofilms.
- Toxicity assessment. Fewer studies directly used biofilm-colonized microplastics to explore their toxicity mechanisms in real environments, whether the biofilm originates from excretion by organisms or natural environmental sources. There is a pressing need to develop more scientifically rigorous methodologies to comprehensively understand the actual ecological risks posed by biofilm-colonized microplastics and other environmental contaminants. Future investigations should also explore the bioavailability of other pollutants absorbed by microplastic biofilms under real exposure scenarios. When microplastics coexist with other pollutants, biofilm formation may not necessarily increase the bioaccumulation of microplastics and their corresponding ecological risks. Thus, it is necessary to investigate the residence duration of biofilm-colonized microplastics in organisms, the bioavailability of exogenous contaminants, and their subsequent fate and potential risks following excretion across extended temporal and varied environmental media.
- Aging and degradation of microplastics. Research has revealed that beyond comprehending the dynamic interplay between microplastics and biofilms from a risk assessment standpoint, biofilms can also serve in the selection of strains for microplastic degradation. Nevertheless, this approach necessitates stringent conditions for microplastics enrichment and currently faces limitations in its practical application for plastic pollution management and control. Exploring more effective approaches to form biofilms and leverage their advantages for controlling plastic pollution could emerge as a focal point for future research and exploration.
